# Ag Atom Anchored on Defective Hexagonal Boron Nitride Nanosheets As Single Atom Adsorbents for Enhanced Adsorptive Desulfurization via S-Ag Bonds

**DOI:** 10.3390/nano12122046

**Published:** 2022-06-14

**Authors:** Hui Liu, Jie Yin, Jinrui Zhang, Hongshun Ran, Naixia Lv, Wei Jiang, Hongping Li, Wenshuai Zhu, Huaming Li

**Affiliations:** 1 Institute for Energy Research, School of Chemistry and Chemical Engineering, Jiangsu University, Zhenjiang 212013, China; lh7544@ujs.edu.cn (H.L.); 15905103795@163.com (J.Y.); 15334557469@163.com (H.R.); jiangwei@ujs.edu.cn (W.J.); lhm@ujs.edu.cn (H.L.); 2 School of the Environment and Safety Engineering, Jiangsu University, Zhenjiang 212013, China; zjr199573@163.com; 3 College of Biology and Chemistry, Xingyi Normal University for Nationalities, Xingyi 562400, China; xiaoxia791102@163.com

**Keywords:** single atom absorbents, Ag atom, adsorptive desulfurization, hexagonal boron nitride, density functional theory

## Abstract

Single atom adsorbents (SAAs) are a novel class of materials that have great potential in various fields, especially in the field of adsorptive desulfurization. However, it is still challenging to gain a fundamental understanding of the complicated behaviors on SAAs for adsorbing thiophenic compounds, such as 1-Benzothiophene (BT), Dibenzothiophene (DBT), and 4,6-Dimethyldibenzothiophene (4,6-DMDBT). Herein, we investigated the mechanisms of adsorptive desulfurization over a single Ag atom supported on defective hexagonal boron nitride nanosheets via density functional theory calculations. The Ag atom can be anchored onto three typical sites on the pristine h-BN, including the monoatomic defect vacancy (B-vacancy and N-vacancy) and the boron-nitrogen diatomic defect vacancy (B-N-divacancy). These three Ag-doped hexagonal boron nitride nanosheets all exhibit enhanced adsorption capacity for thiophenic compounds primarily by the S-Ag bond with π-π interaction maintaining. Furthermore, from the perspective of interaction energy, all three SAAs show a high selectivity to 4,6-DMDBT with the strong interaction energy (−33.9 kcal mol^−1^, −29.1 kcal mol^−1^, and −39.2 kcal mol^−1^, respectively). Notably, a little charge transfer demonstrated that the dominant driving force of such S-Ag bond is electrostatic interaction rather than coordination effect. These findings may shed light on the principles for modeling and designing high-performance and selective SAAs for adsorptive desulfurization.

## 1. Introduction

The rapid acceleration of industrialization has contributed to the increasing demand for diesel fuel. Nevertheless, the combustion of sulfur contents in diesel fuel will generate SO*x*, which seriously poses a threat to the environment on which human beings depend [1,2,3]. Toward this end, strict standards for the sulfur contents in diesel fuel have been established. Conventionally, the hydrodesulfurization (HDS) technique is commonly employed in industry to effectively remove sulfur compounds such as mercaptans and thioethers from diesel fuel, but thiophenic compounds (THs), especially 4,6-Dimethyldibenzothiophene (4,6-DMDBT), cannot be totally removed by this method [4,5,6,7]. Consequently, it is desirable to find an alternative process of non-hydrodesulfurization that can complement HDS.

Adsorptive desulfurization (ADS) is considered to be one of the most promising techniques for industrialization due to its mild conditions and low energy consumption [8,9,10]. In recent years, owing to the excellent thermal stability and large specific surface area, two-dimensional hexagonal boron nitride (h-BN), with a similar structure to graphene, has been widely implemented in the field of adsorption and catalysis [11,12,13,14,15,16]. Quantum chemical calculations have shown that the presence of π-electrons on the surface of h-BN can create strong π-π interactions with THs, thus making it high-performance in the ADS process [17]. On account of the structural similarity between THs and aromatics, adsorbents able to adsorb THs are naturally capable of adsorbing aromatics as well, thus seriously limiting the selectivity of h-BN materials.

To date, metal-organic frameworks as adsorbents have been applied in the field of ADS for the rich pore structure and large specific surface area [18,19,20]. Metal elements are responsible for the strong S-M interactions with S atoms in THs, which is attributed to the lone-pair electrons of S atoms interacting with *s* orbitals in metals [21]. In contrast, aromatics are unable to form this special interaction in the absence of S atoms compared to THs. However, the above materials suffer from low metal utilization, poor regeneration performance, and low ADS performance. For the sake of further improving the ADS performance and reducing the cost, our research emphasis has turned to “single atom adsorbents” (SAAs) by narrowing down the adsorbent to the atomic scale, which provides a platform for new basic science and excellent adsorbent design.

Previous work showed that changing the electronic structure of h-BN is an effective approach [22,23,24]. Furthermore, h-BN can deliver abundant N or B ligands for metal atoms to form stable SAAs. With abundant defect sites and a large specific surface area, h-BN presents an ideal carrier for single atom metal materials [25]. For example, it is reported that metal (Cu, Ag, Au, Pt, Rh, Pd, Fe, Co, and Ir) doped h-BN nanosheets should be stable under high temperatures and possessed catalytic activity for CO oxidation by first-principles computations [26]. Lu et al. discussed the mechanism of CO oxidation to CO_2_ over single Ag atoms supported on h-BN nanosheets by density functional theory (DFT) calculations [27]. Additionally, the oxidation of C_2_H_4_ catalyzed by an Ag atom embedded in h-BN nanosheets was studied by Esrafili et al. [28]. It is foreseeable that h-BN loaded single-atom metal materials could greatly promote ADS reactivity and selectivity.

In this work, we have systematically investigated the ADS performance for THs and aromatics of three SAAs with defective h-BN nanosheets doped with Ag atoms, including boron vacancy, nitrogen vacancy, and boron-nitrogen diatomic vacancy, using DFT calculations. In terms of interaction energy, the ADS performance of all three SAAs was enhanced compared to the pristine h-BN. The Wiberg bond index analysis shows that the S atoms in THs have bonded with Ag atoms, exhibiting the selectivity of SAAs for THs. In addition, a little charge transfer indicates that the main driving force of such S-Ag bond is electrostatic interaction. It is hoped that this work provides a theoretical basis for the rational design of SAAs and the optimization of their adsorption performance.

## 2. Computational Details

### 2.1. Theory

All the structures in the current work were optimized using M06-2X functional with the dispersion-corrected term (M06-2X + D3) at the 6-311G(d,p) level implemented in Gaussian 16 program [29]. The M06-2X functional has proven to be more accurate than B3LYP in main-group thermochemistry, kinetics, noncovalent interactions, and electronic spectroscopy [30]. Therefore, the Minnesota hybrid meta density functional was selected to explore chemical properties and the Grimme’s empirical dispersion correction [31] method was also carried out for more accuracy. For the B, N, and H atoms, the triple-zeta basis set with polarized function (6-311G(d,p)) is an all-electron basis set that was chosen to describe the electronic wave function. Notably, for the Ag-doped species, the coordinate of Ag atom was optimized by the SDD pseudopotential basis set [32].

### 2.2. Model

**Figure 1:**
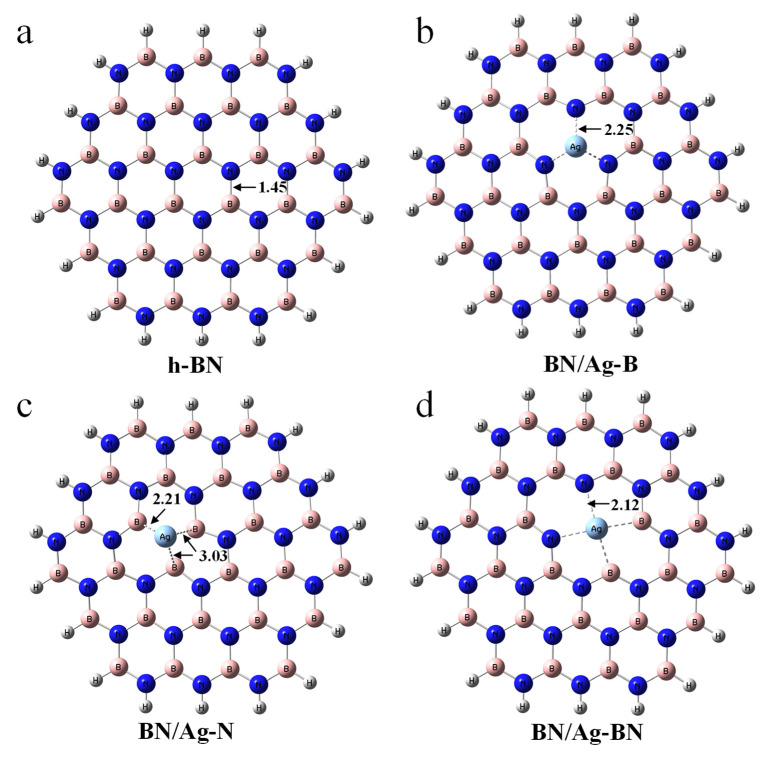
(**a**) The h-BN cluster model (BN_353_). (**b**) The h-BN with the Ag embedded in the B-vacancy defect. (**c**) The h-BN with the Ag embedded in the N-vacancy defect. (**d**) The h-BN with the Ag embedded in the B-N-divacancy defect. B, pink; N, blue; H, white.

**Figure 2:**
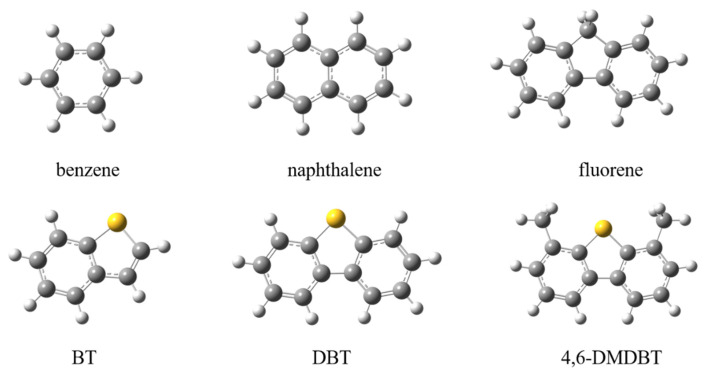
The aromatics and thiophenic compounds used in the present work. S, yellow; C, grey; H, white.

The pristine h-BN monolayer was described by the hydrogen-saturated cluster (B_21_N_21_H_16_), as is shown in Figure 1a, which has been successfully employed to study the non-covalent interaction [17] Three types of defects were studied, including the monoatomic defect vacancy (B-vacancy and N-vacancy) and the boron-nitrogen diatomic defect vacancy (B-N-divacancy). The SAAs, including BN/Ag-B, BN/Ag-N, and BN/Ag-BN, are shown in Figure 1b–d. Furthermore, the adsorbates have been displayed in Figure 2, such as aromatics (benzene, naphthalene, fluorene) and THs (BT, DBT, 4,6-DMDBT).

To evaluate the capacity of Ag-doped species for ADS performance, the interaction energy was obtained as the Equation (1):

(1) $$E_{int}=E_{opt}-E_{adsorbate}-E_{adsorbent}+E_{BSSE}$$

Among them, $E_{opt}$ means the energy of an adsorption complex. $E_{adsorbate}$ represents the energy of the aromatics or thiophenic compounds and $E_{adsorbent}$ is the energy of h-BN or Ag-doped h-BN. Furthermore, $E_{BSSE}$ stands for the basis set superposition error (BSSE) at current calculational level.

## 3. Results and Discussion

### 3.1. Monoatomic Ag-Doped h-BN

Surface vacancies of supporting materials, as previously stated, have great potential to serve as excellent anchoring sites to trap single atoms [33]. The vacancies of h-BN with dangling bonds were believed to function as a support for SAAs for desulfurization. In the first place, to study the ADS performance of the monoatomic nanosheet, the geometric stability of an Ag adatom over h-BN must be verified. Toward this end, the adsorption structures of the Ag atom over three typical sites on the pristine h-BN were considered. Three h-BN nanosheets including B-vacancy, N-vacancy, and B-N-divacancy doped with an Ag atom are named BN/Ag-B (Figure S1), BN/Ag-N (Figure S2), and BN/Ag-BN (Figure S3), respectively. The geometric stability is known to be extremely reliant on the spin multiplicity in the molecular system. Therefore, it is necessary to give priority to the spin multiplicity of Ag-doped h-BN. For BN/Ag-B in Figure S1 and BN/Ag-BN in Figure S3, with the increase of spin multiplicity, the distance between the Ag atom and the h-BN nanosheet goes up gradually. BN/Ag-N nanosheets with different spin multiplicity in Figure S2 present a large deformation. In Table S1, BN/Ag-B_3 have the lowest energy and the structures with a relatively smaller spin multiplicity (BN/Ag-N_1 and BN/Ag-BN_2) as expected are the most stable ones. As a result, great emphasis has been attached to BN/Ag-B_3, BN/Ag-N_1, and BN/Ag-BN_2. As the radius of the Ag atom is greater than that of B and N atoms, the Ag atom in BN/Ag-B_3 and BN/Ag-N_1 thus prefers to lie above the h-BN, as is depicted in Figures S1b and S2a. For the BN/Ag-BN_2 nanosheet in Figure S3a, the B-N-divacancy is large enough to accommodate the Ag atom. Consequently, the structure of BN/Ag-BN_2 (Figure S3a) converges to the plane, compared with BN/Ag-B_3 (Figure S1b) and BN/Ag-N_1 (Figure S2a). Note that BN/Ag-B_3, BN/Ag-N_1, and BN/Ag-BN_2 were selected as monoatomic Ag-doped h-BN nanosheets and were renamed as BN/Ag-B (Figure 1b), BN/Ag-N (Figure 1c) and BN/Ag-BN (Figure 1d), respectively.

In the case of the B-vacancy in Figure 1b, the Ag atom was found to lie at the center of the defect with three equivalent Ag−N bonds (2.25 Å), the Ag atom donates the same electrons to its three neighboring N atoms. Nevertheless, in the case of the N-vacancy in Figure 1c, the distances of Ag-B are 2.21 Å, 3.03 Å, and 3.03 Å, respectively. The Ag atom is favored over the B25 atom to acquire a stronger ionic interaction, rather than being centrally located in the center of N-vacancy. In Figure 1d, the Ag atom is located at the center of the four atoms with four equivalent bonds (2.12 Å). As reported, the formation of a B-vacancy defect in h-BN is more energetically favorable than that of an N-vacancy [25]. Usually, B atoms are positively charged because the electronegativity of N atoms is stronger than that of B atoms, while the Ag atom loses charge in the real bulk with positive charge because of the arrangement of Ag atom valence electrons (4d^10^5s^1^). Therefore, an isolated Ag atom prefers to stably be anchored on the B-vacancy in h-BN rather than N-vacancy.

### 3.2. Adsorption Complexes of Ag-Doped h-BN Nanosheets and Aromatics or Thiophenic Compounds

#### 3.2.1. Structures

h-BN. To investigate the ADS performance of Ag-doped h-BN nanosheets, we first evaluate the adsorption capacity of the pristine h-BN monolayer. All the THs and aromatics in Figure S4 are parallel to the h-BN layer via π-π interaction with a vertical distance of ~3.2 Å [22]. The C atoms in the aromatics tend to be located vertically above the B atom. In addition, the centers of the selected aromatics can be observed against the N atoms.

**Figure 3:**
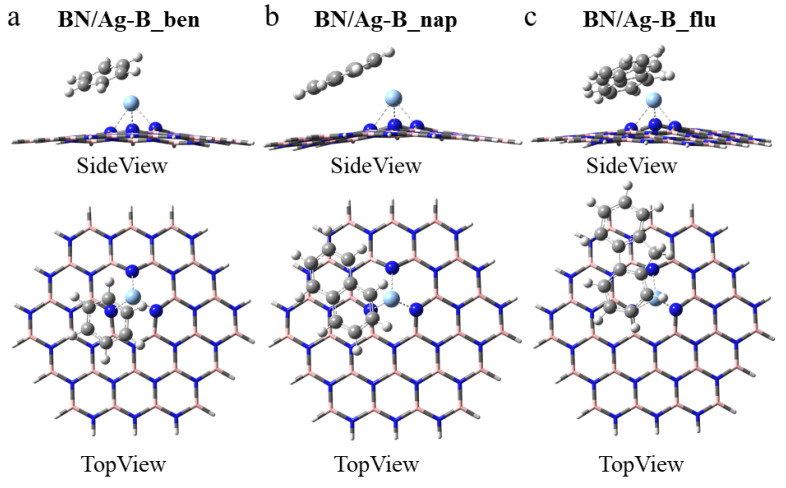

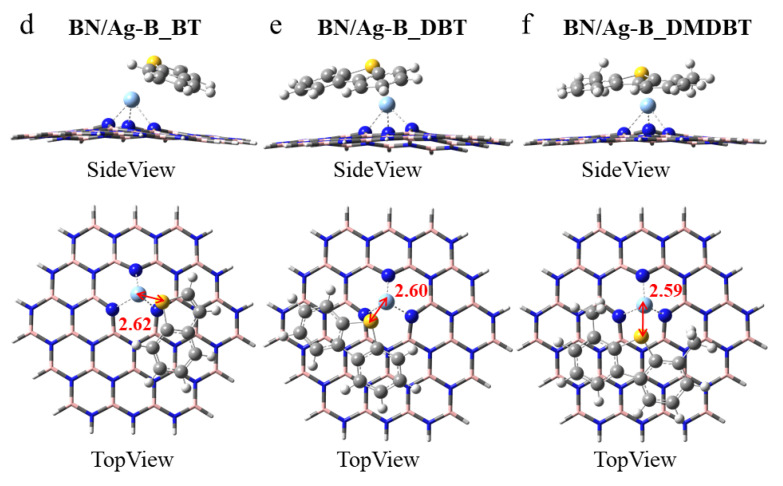
Optimized monoatomic h-BN with the Ag embedded in the B-vacancy defect for adsorbing (**a**) benzene, BN/Ag-B_ben, (**b**) naphthalene, BN/Ag-B _nap, (**c**) fluorene, BN/Ag-B_flu, (**d**) BT, BN/Ag-B_BT, (**e**) DBT, BN/Ag-B_DBT, and (**f**) 4,6-DMDBT, BN/Ag-B_DMDBT.

BN/Ag-B. In contrast with the pristine h-BN, the Ag-doped h-BN nanosheets after adsorption are no longer flat. Therefore, the adsorption of aromatics by Ag-doped h-BN becomes more complicated. As in Figure 3, all aromatics have to be as parallel as possible to the BN/Ag-B on the one hand, and the surface in benzene, naphthalene and fluorene seems to be attached to the Ag atoms on the other hand. As in Figure 3d–f, the S-Ag bond lengths are 2.62 Å, 2.60 Å, and 2.59 Å, respectively. The reduced average bond length implies that BN/Ag-B has a stronger adsorption capacity for 4,6-DMDBT. After adsorption, the Ag atom originally in the center of B-vacancy moved slightly away from h-BN with an increase in distance. In Table S2, the three Ag-N bond lengths do not lie on equal. The average Ag-N bond length is smaller after the adsorption of THs on BN/Ag-B compared to aromatics.

**Figure 4:**
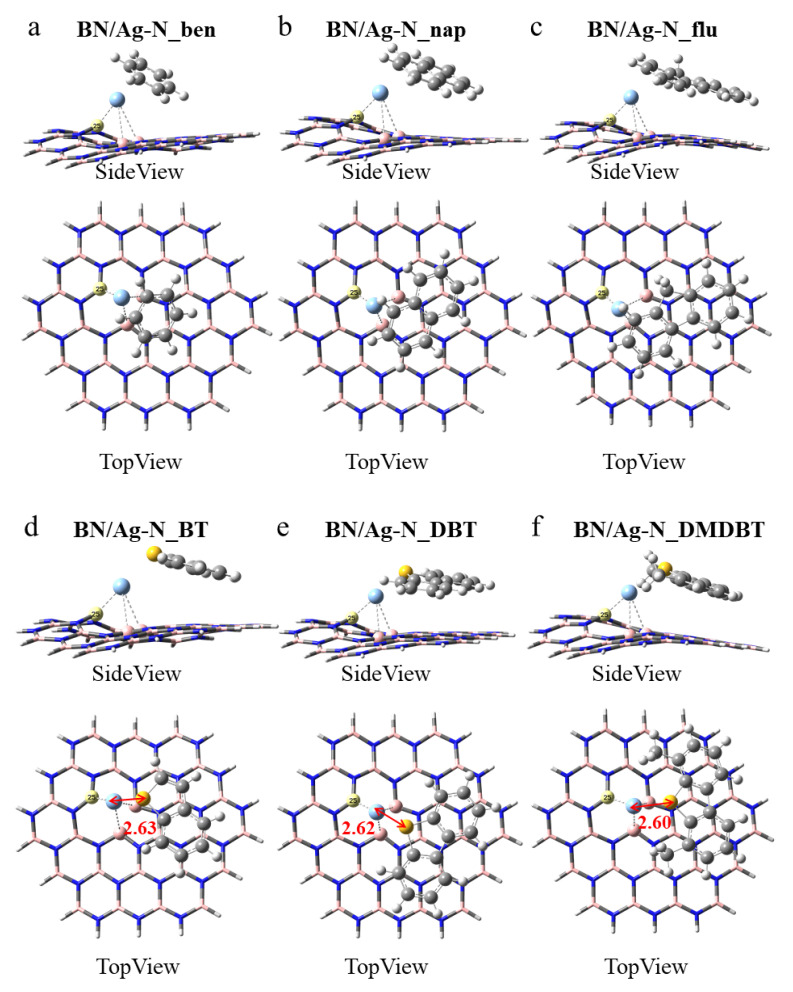
Optimized monoatomic pristine h-BN with the Ag embedded in the N-vacancy defect for adsorbing (**a**) benzene, BN/Ag-N_ben, (**b**) naphthalene, BN/Ag-N_nap, (**c**) fluorene, BN/Ag-N_flu, (**d**) BT, BN/Ag-N_BT, (**e**) DBT, BN/Ag-N_DBT, and (**f**) 4,6-DMDBT, BN/Ag-N_DMDBT. The highlighted atom is the boron atom labeled number 25.

BN/Ag-N. As shown in Figure 4, the BN/Ag-N nanosheet is significantly more deformed than the BN/Ag-B nanosheet in Figure 3. Aromatics should likewise be as parallel to BN/Ag-N as possible to achieve the most stable conformation (Figure 4a–c). As depicted in Figure 4d–f, the distance of S atom and Ag atom after adsorption of THs is 2.63 Å, 2.62 Å, and 2.60 Å, respectively. Compared with the S-Ag bond length in Figure 3, the values are all slightly larger, indicating weaker interaction of the BN/Ag-N nanosheet. In Table S2, the Ag-B25 bond length equals 2.20 Å in all conformations, which means a strong bond has formed between the Ag atom and the B25 atom. The Ag-B average bond lengths in BN/Ag-N_DBT and BN/Ag-N_DMDBT are both 2.80 Å, but the S-Ag bond length in BN/Ag-N_DMDBT is shorter, implying BN/Ag-N nanosheet is more favorable to adsorb 4,6-DMDBT. What’s more, the average Ag-B bond length in BN/Ag-N_DMDBT is smaller than that of BN/Ag-N_flu, presumably attributed to the strong attraction of S-Ag bond.

**Figure 5:**
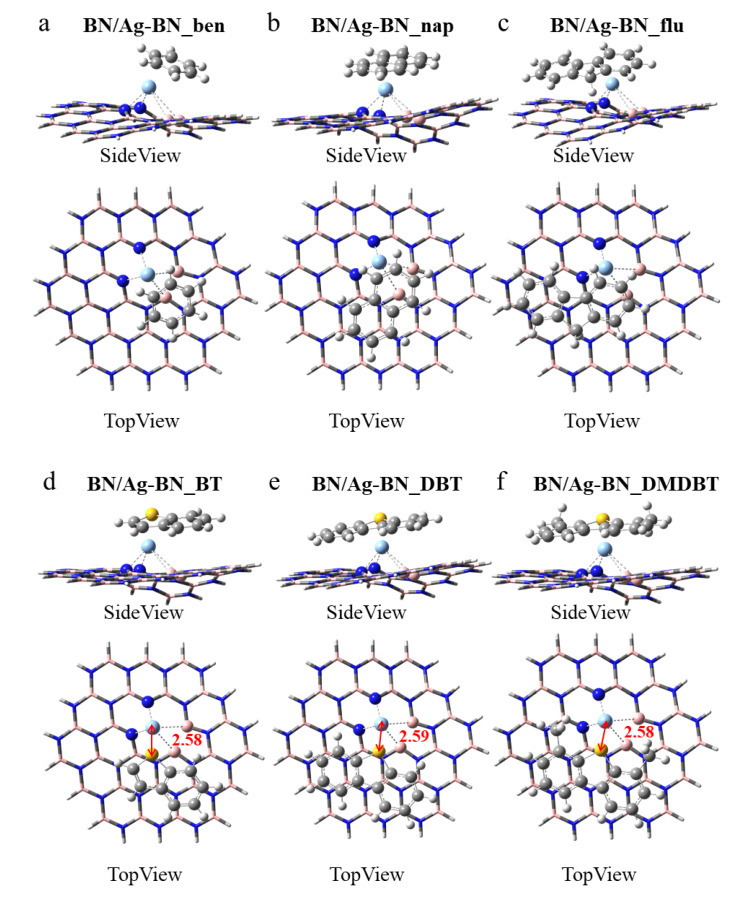
Optimized diatomic h-BN with the Ag embedded in the B-N-divacancy defect for adsorbing (**a**) benzene, BN/Ag-BN_ben, (**b**) naphthalene, BN/Ag-BN _nap, (**c**) fluorene, BN/Ag-BN_flu, (**d**) BT, BN/Ag-BN_BT, (**e**) DBT, BN/Ag-BN_DBT, and (**f**) 4,6-DMDBT, BN/Ag-BN_DMDBT.

BN/Ag-BN. Interestingly, the BN/Ag-BN adsorption of aromatics generated severe deformation, as displayed in Figure 5a–c. For the THs of BN/Ag-BN, the S-Ag bond lengths of BT (Figure 5d), DBT (Figure 5e), and 4,6-DMDBT (Figure 5f) are only ~2.58 Å, which was smaller than those in BN/Ag-B and BN/Ag-N, demonstrating that the B-N-divacancy was much more capable of adsorbing THs. In Table S2, the Ag-B average bond lengths of THs are small, compared to those of the corresponding aromatics.

#### 3.2.2. Energetics

An overview of the interactions of aromatics and thiophenic compounds with various nanosheets has been provided roughly by structure analysis, but it is difficult to grasp the ADS performance integrally. Herein, $E_{int}$ was calculated in Table 1 and Table S3 to understand the strength of the adsorption capacity. As reported, 4,6-DMDBT is hard to be totally removed because it is considered as resistant to chemical reactions [7]. Hence, it is necessary to put emphasis on efficiently removing 4,6-DMDBT. In the first place, the pristine h-BN is taken as a comparison (Table S3) to investigate whether Ag-doped h-BN enhances the ability of ADS. It is well known that π-π interaction plays an important role in the adsorption process of two-dimensional materials [17]. Therefore, for the pristine h-BN, π-π interaction is the main force to adsorb aromatics and THs with a poor adsorption capacity. To be specific, the $E_{int}$ of benzene, naphthalene, and fluorene (or BT, DBT and 4,6-DMDBT) on the pristine h-BN are −10.4 kcal mol^−1^, −16.7 kcal mol^−1^, and −20.8 kcal mol^−1^ (−15.2 kcal mol^−1^, −20.8 kcal mol^−1^, and −22.0 kcal mol^−1^), respectively. Among them, the $E_{int}$ of fluorene and 4,6-DMDBT are relatively similar and difficult to separate selectively using the pristine h-BN. Fortunately, the adsorption capacity of SAAs in Table 1, especially BN/Ag_BN, is significantly improved. The BN/Ag-B shows stronger adsorption abilities with BT, DBT, and 4,6-DMDBT than THs’ corresponding aromatics, which was attributed to the S-Ag bond. Generally, the S-Ag bond length is proportional to the $E_{int}$. In Table S4, the $E_{int}$ and the S-Ag bond length of the thiophenic sulfides follows the order BN/Ag-N > BN/Ag-B > BN/Ag-BN. The $E_{int}$ of BN/Ag-B_DMDBT (−33.9 kcal mol^−1^) is much stronger than that of BN/Ag-B_flu (−25.3 kcal mol^−1^), indicating a strong selectivity of BN/Ag-B for 4,6-DMDBT. In addition, BN/Ag-N prefers to adsorb 4,6-DMDBT (−29.1 kcal mol^−1^). For BN/Ag-BN, the $E_{int}$ of BT, DBT and 4,6-DMDBT on BN/Ag-BN are −28.1 kcal mol^−1^, −34.4 kcal mol^−1^, 39.2 kcal mol^−1^, respectively, demonstrating that BN/Ag-BN has a significant boost for adsorbing THs. Such diatomic defective site doped Ag atom is more favorable for the removal of THs from fuel, especially for 4,6-DMDBT. In short, ascribed to the S-Ag bond, SAAs have a superior adsorption capacity for THs and exhibits selectivity for 4,6-DMDBT. SAAs formed by doping Ag atom onto defective sites is expected to selectively remove specific-target THs.

### 3.3. Analysis of the Nature of Thiophenic Compounds Adsorption

Charge analysis. Natural population analysis (NPA) is a method for calculating the atomic charge and orbital population of molecular wave functions in general atomic orbital basis sets [34]. NPA provides a better description of the electronic distribution of compounds containing metal atoms than traditional Mulliken population analysis. Δq is a parameter to describe the charge change of atom or species before and after adsorption. As is in Table 2, the pristine h-BN with Lewis acidic sites and Lewis basic sites can adsorb THs through Lewis acid-base interactions, as expected, resulting in negligible charge change [35]. The values of Δq (THs) in SAAs are larger than those in pristine h-BN on account of the presence of an Ag atom that makes the overall charge of THs transfer to SAAs through the S-Ag bond. After adsorption, the Ag atoms in BN/Ag-B obtain −0.229, −0.233, and −0.245, respectively, with most of the charge coming from THs. Such a large charge transfer is also one of the reasons for the enhanced adsorption capacity. It is noteworthy that the Ag atoms in BN/Ag-N lost electrons instead. In the structure analysis, we found that an Ag-B bond exists in BN/Ag-N. After adsorption of THs, the charge changes of this B atom are −0.088, −0.081, and −0.087, respectively. It may be that the B25 atom with relatively strong electronegativity will acquire the charge gained by the Ag atom from THs, leaving the Ag charge reduced after adsorption. Furthermore, the values of Δq (THs) in BN/Ag-N_DMDBT are small, resulting in a weaker adsorption capacity. For BN/Ag-BN, the changes in both S and Ag are quite marginal, suggesting little charge transfer between the S atom and Ag atom. Furthermore, Δq (THs) are large, demonstrating an enhanced adsorption capacity. Overall, the Δq (THs) in SAAs is a significant indicator of the enhanced adsorption capacity compared to the pristine h-BN. What’s more, the smaller Δq (S) indicates a little charge transfer between the Ag and S.

Wiberg bond index analysis. When it comes to the structural properties of a compound, we often need to know the bonding properties between atoms, one of the more important ones being the bond order. The Wiberg bond index (WBI) is calculated as the quadratic non-diagonal sum of elements atoms of the density matrix between two atoms [36]. Here, WBI has been used to evaluate the strength between S and Ag atom, which has been employed in a similar system before [37]. The distance between S and Ag is ~2.6 Å, which is smaller than their van der Waals radius (4.42 Å) [38], demonstrating a strong interaction. In Table 3, strong S-Ag bonds came into existence with a WBI greater than 0.22. Furthermore, the WBI in BN/Ag-B and BN/Ag-BN is generally greater than that in BN/Ag-N, accounting for the enhanced adsorption of BN/Ag-B and BN/Ag-BN. For 4,6-DMDBT, the WBI of BN/Ag-B_DMDBT (0.3255), BN/Ag-N_DMDBT (0.3093), BN/Ag-BN_DMDBT (0.3118) are all greater than 0.3, demonstrating a strong interaction between S and Ag atom in 4,6-DMDBT. Overall, considering a little charge transfer in NPA, it can be safely concluded that the main driving force of S-Ag bonds is electrostatic interaction.

**Figure 6:**
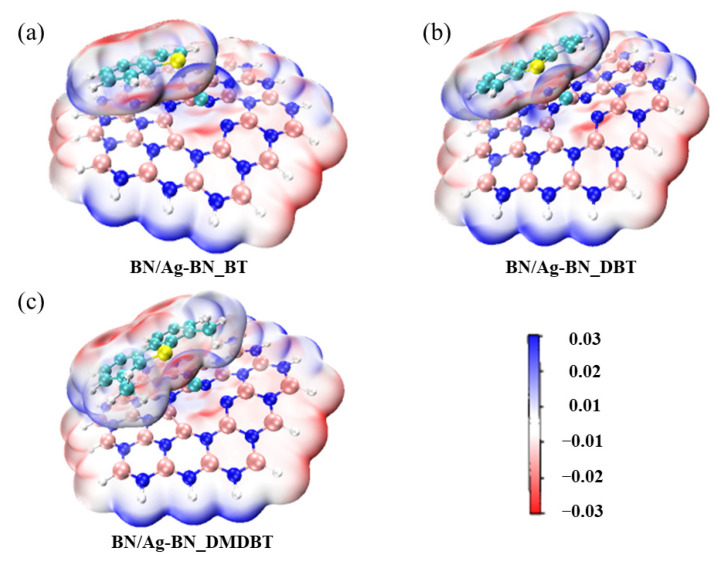
Electrostatic potential analysis results on 0.001 a.u. contours of the electronic density for the BN/Ag-BN after adsorption (positive regions are indicated in blue, and negative regions are indicated in red). (**a**) BN/Ag-BN_BT, (**b**) BN/Ag-BN_DBT, (**c**) BN/Ag-BN_DMDBT.

Electrostatic potential analysis. The electrostatic potential analysis is an effective tool to predict the interaction sites [39]. The electrostatic potential is one of the important physical properties of molecules that defines the work carried out to move a unit positive charge from infinity to a point in space around the molecule [40]. As many atoms constitute a molecule, the molecular charge distribution is no longer uniform but has positive and negative potential regions due to the difference in electronegativity of individual atoms [41]. Low-density regions often have a positive electrostatic potential plotted in blue, while high-density regions present a negative electrostatic potential painted in red. The theoretical basis is that molecules always tend to approach each other in a complementary manner to ESP [42]. As suggested by Bader et al., we selected the isosurface (isovalue = 0.001) with the charge containing approximately 97% of the molecule [43]. It is universally known that the electronegativity of N atoms is higher than that of B atoms, so in the pristine h-BN (Figure S5a), the positively charged regions are concentrated on B atoms, while the negatively charged regions are focused on N atoms. Nevertheless, the conjugation effect makes the charge regions on the surface of the pristine h-BN equalized. The addition of Ag makes the surface charge of defective h-BN no longer evenly distributed, which allows SAAs more reactive. As shown in Figure S5b, the surfaces of the selected aromatics in this work are all negatively charged, because the surrounding H atoms are electron-donating groups that make the C and S atoms negatively charged. Hence, after adsorption, the negatively charged center of the aromatics will be attracted to positively charged B atoms in h-BN nanosheets. In addition, the S atoms tend to be located near the Ag atom to form the S-Ag bond. In order to prove whether there exists electrostatic interaction between S and Ag atoms, the intermolecular van der Waals surface was plotted in Figure 6 using VMD [44] program. The electrostatic potential involved in the analyses was evaluated by Multiwfn [45] based on the highly effective algorithm proposed in Ref. [46]. The positive surface potential of the Ag atom overlaps with the negative surface potential of the S atom. The large overlap implies a strong electrostatic interaction between Ag and S atoms, which agrees well with the results of NPA and WBI analysis. In the ADS process utilizing SAAs, with the dominance of dispersive interactions (π-π), the electrostatic interaction between Ag and benzene ring enhances the adsorption capacity, while S-Ag bonds strengthen the selectivity for THs.

Natural bond orbital analysis. The natural bond orbital analysis provides a useful means for examining charge delocalization and conjugative interactions in the current system [47]. To investigate the acceptor-donor interactions in S-Ag bonding arrays quantitatively, the second-order perturbation approach is a method for studying the interaction between ‘‘donor” Lewis-type NBOs and ‘‘acceptor” non-Lewis NBOs. For each donor (*i*) and acceptor (*j*) natural bond orbital, the stabilization energy (hyperconjugative interaction energy) between donor and acceptor was reflected by the value of E(2) calculated as follows:

(2) $$E(2)=△E_{ij}=q_{i}\frac{F^{2}\left(i,j\right)}{\varepsilon_{i}-\varepsilon_{j}}$$

where $q_{i}$ is the occupancy of the donor orbital, $\varepsilon_{i}$ and $\varepsilon_{j}$ are diagonal elements (i.e., orbital energies) in the NBO Fock matrix and F(i,j) is the off-diagonal Fock matrix element between *i* and *j* NBO orbitals.

**Figure 7:**
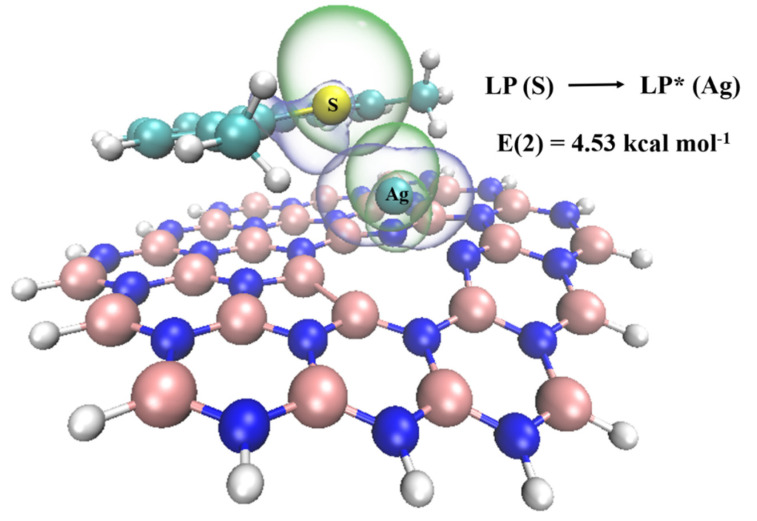
The interacting donor and acceptor orbitals in BN/Ag-BN_DMDBT.

The larger stabilization energy E(2) between the electron-donating bond orbital and the electron-accepting anti-bonding orbital corresponds to stronger interaction and greater hyperconjugation in multiple. Remarkably, the overlap between the orbitals of the S atom and Ag atom can be observed in Figure 7. The stabilization energy between the lone pair (LP) of electrons in the S atom and the antibonding orbital (LP*) in the Ag atom reached 4.53 kcal mol^−1^. Remarkably, LP* refers to the antibonding orbital dominated by the 5 s orbital in the Ag atom. Consequently, there exists strong interaction between S and Ag atom.

**Figure 8:**
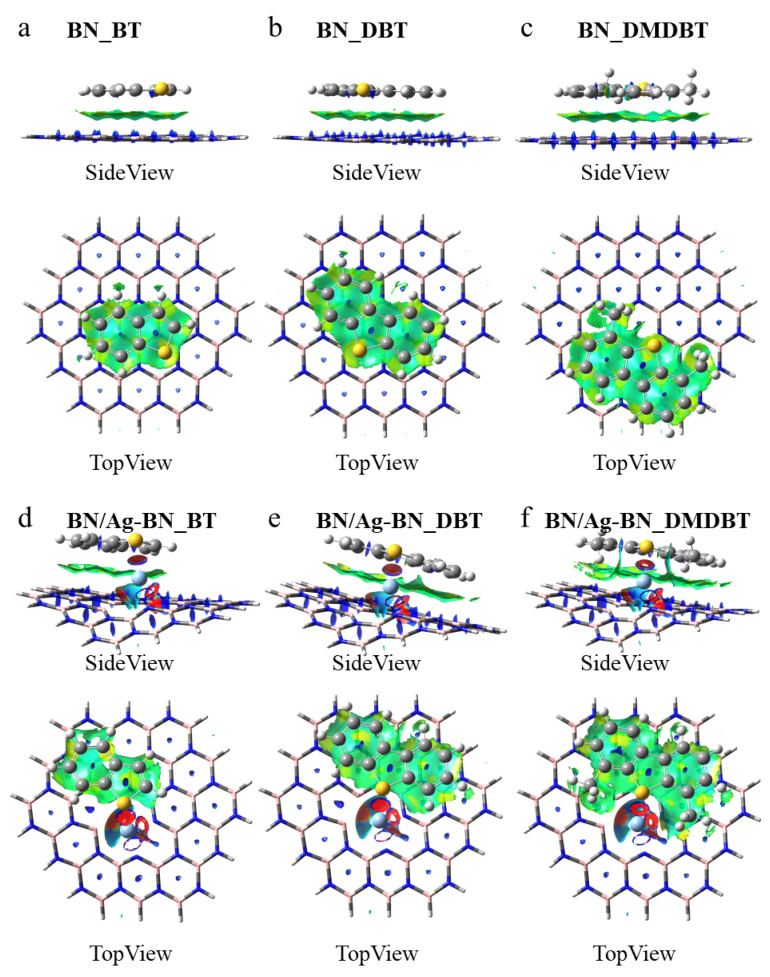
Gradient isosurfaces (s = 0.5 a.u.) of the pristine h-BN and BN/Ag-BN nanosheets for adsorption. The surfaces are colored on a red-green-blue scale according to values of sign (λ_2_)ρ, ranging from −0.02 to 0.02 a.u. Red means strong attractive interactions and blue indicates strong nonbonded overlap. (**a**) BN_BT, (**b**) BN_DBT, (**c**) BN_DMDBT, (**d**) BN/Ag-BN_BT, (**e**) BN/Ag-BN_DBT, (**f**) BN/Ag-BN_DMDBT.

Reduced density gradient analysis. Given atomic interactions are ubiquitous in chemical systems, the graphical representation of interactions is valuable for examining chemical issues, allowing chemists to quickly visualize the types of interactions and their specific positions in a system. The reduced density gradient method proposed by Yang et al. is a method to visualize the types of interactions by plotting the reduced density gradient versus the electric density multiplied by the sign of the second Hessian eigenvalue [48]. It reveals the basic chemistry that makes up covalent structures and provides a rich representation of van der Waals interactions, hydrogen bonding, and spatial repulsion in small molecules, molecular complexes, and solids. Red indicates strong attractive interactions (e.g., hydrogen bonding and strong electrostatic interactions), transition regions indicate typical van der Waals interactions, and blue represents strong non-bonding overlap [49]. As in Figure 8 and Figure S6, the typical van der Waals interaction in green between the pristine h-BN and THs is the π-π interaction. However, for the Ag-doped h-BN, in addition to the large region in green, there remains a red region between Ag and S atom, indicating a stronger bond, which is consistent with the previous analysis. In addition, there exists a strong interaction between Ag and the surrounding B or N atoms, implying that Ag can be anchored to the defective h-BN nanosheets. Overall, SAAs exhibit enhanced ADS selectivity through S-Ag bonds with π-π interaction dominated.

## 4. Conclusions

In this work, we comprehensively investigated the mechanism behind the selectivity and enhanced ADS performance of SAAs by doping Ag onto h-BN nanosheets. The defective h-BN nanosheets promise an ideal carrier for single atom metal adsorbents. The SAAs, including BN/Ag-B, BN/Ag-N, and BN/Ag-BN, shows enhanced adsorption capacity for THs primarily through the S-Ag bond while the π-π interaction is maintained. What’s more, all the three SAAs have a high selectivity to 4,6-DMDBT with a strong interaction energy (−33.9 kcal mol^−1^, −29.1 kcal mol^−1^, and −39.2 kcal mol^−1^, respectively). The quantum chemical analysis demonstrates that the main driving force of such S-Ag bond is electrostatic interaction because of little charge transfer, which is responsible for the selectivity and promotion of ADS performance for THs.

## Figures and Tables

**Table 1:** Interaction energy of the defective structures with the Ag embedded for adsorbing aromatics and thiophenic sulfides. (Units: kcal mol^−1^).

Species	$E_{int}$
BN/Ag-B	
BN/Ag-B_ben	−20.6
BN/Ag-B_nap	−24.4
BN/Ag-B_flu	−25.3
BN/Ag-B_BT	−23.3
BN/Ag-B_DBT	−29.1
BN/Ag-B_DMDBT	**−33.9**
BN/Ag-N	
BN/Ag-N_ben	−17.9
BN/Ag-N_nap	−20.3
BN/Ag-N_flu	−22.5
BN/Ag-N_BT	−20.4
BN/Ag-N_DBT	−24.4
BN/Ag-N_DMDBT	**−29.1**
BN/Ag-BN	
BN/Ag-BN_ben	−25.5
BN/Ag-BN_nap	−17.2
BN/Ag-BN_flu	−32.4
BN/Ag-BN_BT	−28.1
BN/Ag-BN_DBT	−34.4
BN/Ag-BN_DMDBT	**−39.2**

**Table 2:** Charge analysis of adsorption complexes. (Units: e).

Species	Δq (S) ^a^	Δq (THs) ^b^	Δq (Ag) ^c^	Δq (Ads) ^d^
h-BN				
BN_BT	0.021	0.051	-	−0.051
BN_DBT	0.024	0.059	-	−0.059
BN_DMDBT	0.026	0.065	-	−0.065
BN/Ag-B				
BN/Ag-B_BT	0.027	0.189	−0.229	−0.189
BN/Ag-B_DBT	0.035	0.198	−0.465	−0.198
BN/Ag-B_DMDBT	0.038	0.226	−0.483	−0.226
BN/Ag-N				
BN/Ag-N_BT	0.005	0.161	0.015	−0.161
BN/Ag-N_DBT	0.022	0.164	0.005	−0.164
BN/Ag-N_DMDBT	0.024	0.193	0.002	−0.193
BN/Ag-BN				
BN/Ag-BN_BT	0.019	0.192	−0.009	−0.192
BN/Ag-BN_DBT	0.027	0.202	−0.013	−0.202
BN/Ag-BN_DMDBT	0.029	0.217	−0.028	−0.217

^a^ Charge change of the S atom. ^b^ Charge change of thiophenic sulfides. ^c^ Charge change of the Ag atom. ^d^ Charge change of the adsorbents.

**Table 3:** The bond length and the WBI of S-Ag bonds.

Species	Bond Length	WBI
BN/Ag-B		
BN/Ag-B_BT	2.62 Å	0.3031
BN/Ag-B_DBT	2.60 Å	0.3108
BN/Ag-B_DMDBT	2.59 Å	0.3255
BN/Ag-N		
BN/Ag-N_BT	2.63 Å	0.2200
BN/Ag-N_DBT	2.62 Å	0.2353
BN/Ag-N_DMDBT	2.60 Å	0.3093
BN/Ag-BN		
BN/Ag-BN_BT	2.58 Å	0.3115
BN/Ag-BN_DBT	2.59 Å	0.2471
BN/Ag-BN_DMDBT	2.58 Å	0.3118

## Data Availability

The data presented in this study are available on request from the corresponding author.

## Supplementary Materials

The following supporting information can be downloaded at: https://www.mdpi.com/article/10.3390/nano12122046/s1, Figure S1: Optimized monoatomic h-BN of different spin multiplicity (S = 1, 3, 5) with the Ag embedded in the B-vacancy defect. (a) BN/Ag-B_1, (b) BN/Ag-B_3, (c) BN/Ag-B_5; Figure S2: Optimized monoatomic h-BN of different spin multiplicity (S = 1, 3, 5) with the Ag embedded in the N-vacancy defect. (a) BN/Ag-N_1, (b) BN/Ag-N_3, (c) BN/Ag-N_5; Figure S3: Optimized monoatomic h-BN of different spin multiplicity (S = 2, 4, 6) with the Ag embedded in the B-N-divacancy defect. (a) BN/Ag-BN_2, (b) BN/Ag-BN_4, (c) BN/Ag-BN_4; Table S1: Relative energy of h-BN and the defective structures with the Ag embedded; Figure S4: Optimized monoatomic h-BN for adsorbing (a) benzene, BN_ben, (b) naphthalene, BN_nap, (c) fluorene, BN_flu, (d) BT, BN_BT, (e) DBT, BN_DBT, and (f) 4,6-DMDBT, BN_DMDBT; Table S2: The bond length of Ag-N or Ag-B in SAAs after adsorption; Table S3: Interaction energy of the pristine h-BN nanosheet for adsorbing aromatics and thiophenic sulfides; Table S4: The relationship between the $E_{int}$ and the S-Ag bond length; Figure S5: Electrostatic potential surface mapped on electron total density with an isovalue of 0.001. The colours range from −0.02 a.u. in red to 0.02 a.u. in blue for all the molecules. (a) The h-BN and the defective structures with the Ag embedded; (b) The aromatic compounds as absorbates; Figure S6: Gradient isosurfaces (s = 0.5 a.u.) of the BN/Ag-B and BN/Ag-N nanosheets for adsorption. The surfaces are colored on a red-green-blue scale according to values of sign (λ2)ρ, ranging from −0.02 to 0.02 a.u. (a) BN/Ag-B_BT, (b) BN/Ag-B_DBT, (c) BN/Ag-B_DMDBT, (d) BN/Ag-B_BT, (e) BN/Ag-B_DBT, (f) BN/Ag-B_DMDBT.
