# Supplementary material for: Ag Atom Anchored on Defective Hexagonal Boron Nitride Nanosheets As Single Atom Adsorbents for Enhanced Adsorptive Desulfurization via S-Ag Bonds
Source: Nanomaterials (Basel). 2022 Jun 14;12(12):2046. doi: 10.3390/nano12122046 (PMC9230516; doi:10.3390/nano12122046)
Supplement: Supplementary file 1 [file nanomaterials-12-02046-s001.zip › nanomaterials-1732381-supplementary.pdf]

nanomaterials

Supplementary Materials

Ag Atom Anchored on Defective Hexagonal Boron Nitride Nanosheets As single Atom Adsorbents for Enhanced Adsorptive Desulfurization via S-Ag Bonds

Hui Liu [1,†], Jie Yin [1,†], Jinrui Zhang [2], Hongshun Ran [1], Naixia Lv [3], Wei Jiang [1], Hongping Li [1,*], Wenshuai Zhu [1,*] and Huaming Li [1]

[1] Institute for Energy Research, School of Chemistry and Chemical Engineering, Jiangsu University, Zhenjiang 212013, China; lh7544@ujs.edu.cn (H.L.); 15905103795@163.com (J.Y.); 15334557469@163.com (H.R.); jiangwei@ujs.edu.cn (W.J.); lhm@ujs.edu.cn (H.L.)
[2] School of the Environment and Safety Engineering, Jiangsu University, Zhenjiang 212013, China; zjr199573@163.com
[3] College of Biology and Chemistry, Xingyi Normal University for Nationalities, Xingyi 562400, China; xiaoxia791102@163.com
* Correspondence: hongpingli@ujs.edu.cn (H.L.); zhuws@ujs.edu.cn (W.Z.)
† These authors contributed equally to this work.

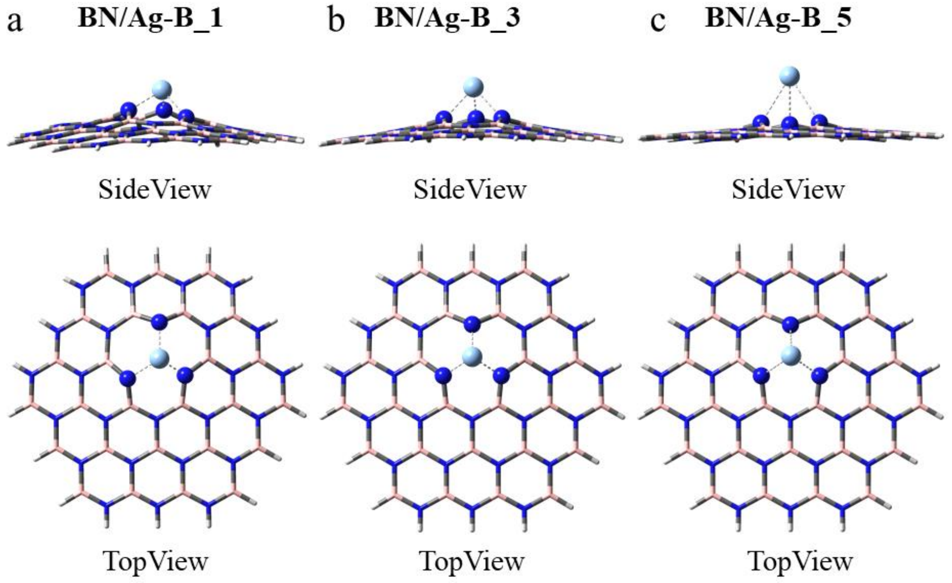

Figure S1. Optimized monoatomic h-BN of different spin multiplicity (S = 1, 3, 5) with the Ag embedded in the B-vacancy defect. (**a**) BN/Ag-B_1, (**b**) BN/Ag-B_3, (**c**) BN/Ag-B_5.

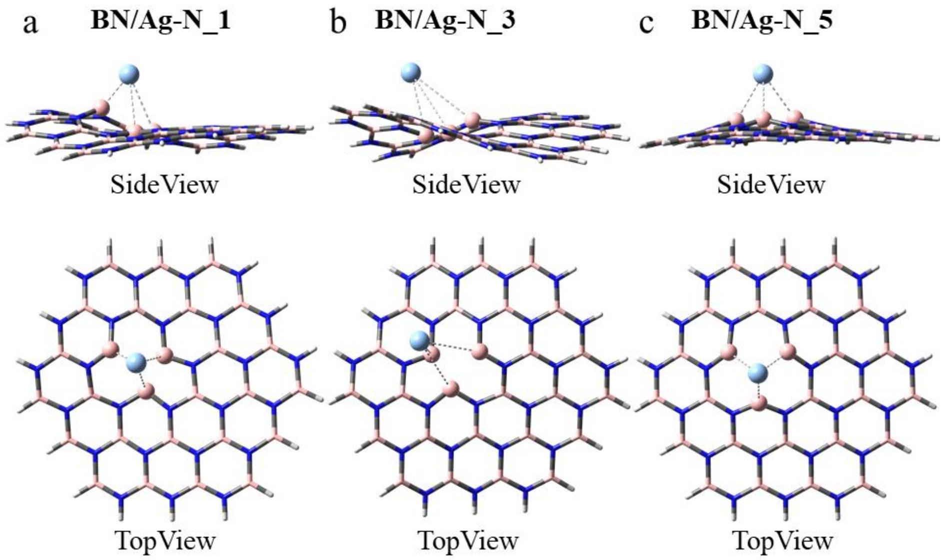

Figure S2. Optimized monoatomic h-BN of different spin multiplicity (S = 1, 3, 5) with the Ag embedded in the N-vacancy defect. (**a**) BN/Ag-N_1, (**b**) BN/Ag-N_3, (**c**) BN/Ag-N_5.

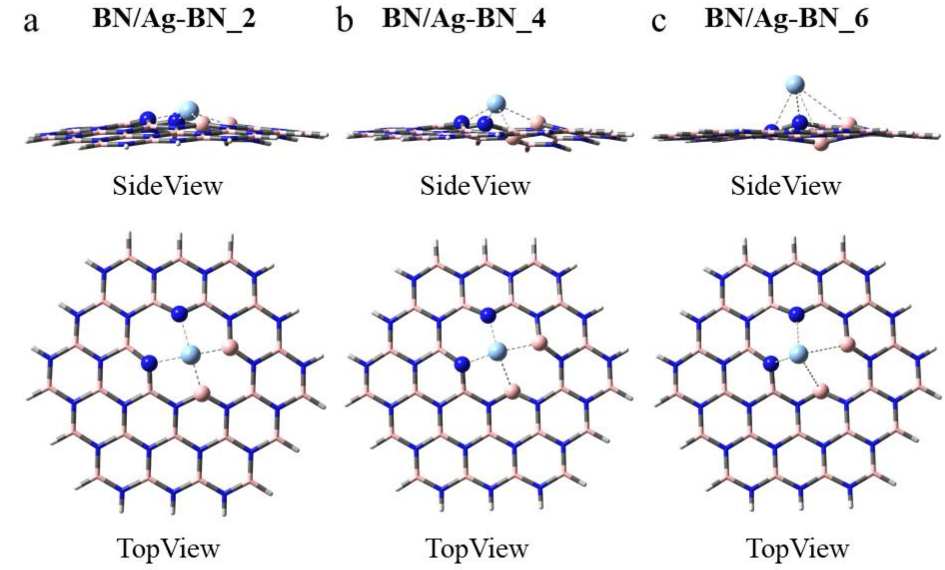

Figure S3. Optimized monoatomic h-BN of different spin multiplicity (S = 2, 4, 6) with the Ag embedded in the B-N-divacancy defect. (**a**) BN/Ag-BN_2, (**b**) BN/Ag-BN_4, (**c**) BN/Ag-BN_4.

Table S1. Relative energy of h-BN and the defective structures with the Ag embedded. (Units: kcal mol^{-1}).

Species	Relative Energy
BN/Ag-B	
BN/Ag-B_1	29.0
**BN/Ag-B_3**	**0**
BN/Ag-B_5	67.8
BN/Ag-N	
**BN/Ag-N_1**	**0**
BN/Ag-N_3	30.2
BN/Ag-N_5	114.5
BN/Ag-BN	

**BN/Ag-BN_2**	**0**
BN/Ag-BN_4	54.5
BN/Ag-BN_6	132.4

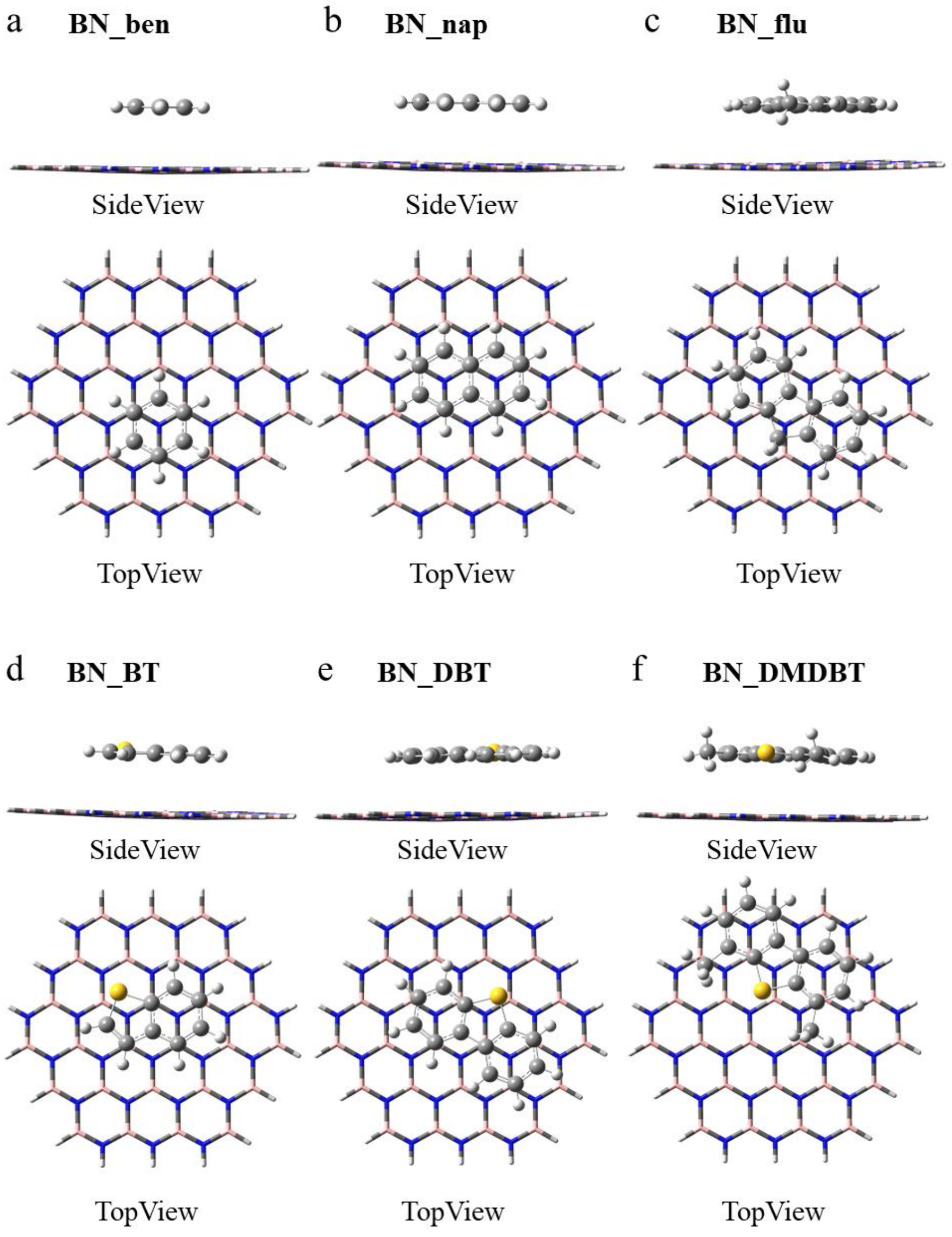

Figure S4. Optimized monoatomic h-BN for adsorbing (**a**) benzene, BN_ben, (**b**) naphthalene, BN_nap, (**c**) fluorene, BN_flu, (**d**) BT, BN_BT, (**e**) DBT, BN_DBT, and (**f**) 4,6-DMDBT, BN_DMDBT.

Table S2. The bond length of Ag-N or Ag-B in SAAs after adsorption.

Species	Bond Length (Å)	Average Bond Length (Å)
**BN/Ag-B**	**Ag-N**	**Ag-N**
BN/Ag-B_ben	2.29, 2.26, 2.35	2.30
BN/Ag-B_nap	2.24, 2.34, 2.36	2.31
BN/Ag-B_flu	2.24, 2.31, 2.37	2.31
BN/Ag-B_BT	2.26, 2.26, 2.33	2.28

BN/Ag-B_DBT	2.31, 2.22, 2.33		2.29	
BN/Ag-B_DMDBT	2.20, 2.30, 2.36		2.29	
BN/Ag-N	Ag-B		Ag-B [a]	
BN/Ag-N_ben	2.20, 3.02, 3.03		2.20, 3.02	
BN/Ag-N_nap	2.20, 2.89, 3.01		2.20, 2.95	
BN/Ag-N_flu	2.20, 2.98, 2.80		2.20, 2.89	
BN/Ag-N_BT	2.20, 2.81, 2.92		2.20, 2.87	
BN/Ag-N_DBT	2.20, 2.80, 2.80		2.20, 2.80	
BN/Ag-N_DMDBT	2.20, 2.78, 2.82		2.20, 2.80	
BN/Ag-BN	Ag-N	Ag-B	Ag-N	Ag-B
BN/Ag-BN_ben	2.28, 2.24	3.00, 3.03	2.26	3.02
BN/Ag-BN_nap	2.23, 2.30	2.93, 3.02	2.27	2.98
BN/Ag-BN_flu	2.22, 2.32	2.96, 2.95	2.27	2.96
BN/Ag-BN_BT	2.32, 2.21	2.98, 2.92	2.27	2.95
BN/Ag-BN_DBT	2.21, 2.34	2.94, 2.88	2.28	2.91
BN/Ag-BN_DMDBT	2.21, 2.35	2.91, 2.88	2.28	2.90

[a] In BN/Ag-N, the average bond length of the latter two has been calculated because the bond length of Ag-B25 equals 2.20 Å.

Table S3. Interaction energy of the pristine h-BN nanosheet for adsorbing aromatics and thiophenic sulfides. (Units: kcal mol^{-1}).

Species	E_{int}
h-BN	
BN_ben	−10.4
BN_nap	−16.7
BN_flu	−20.8
BN_BT	−15.2
BN_DBT	−20.8
BN_DMDBT	−22.0

Table S4. The relationship between the E_{int} and the S-Ag bond length.

Species	E_{int}/(kcal mol^{-1})	S-Ag bond length/(Å)
	**BT**	
BN/Ag-B_BT	−23.3	2.62
BN/Ag-N_BT	−20.4	2.63
BN/Ag-BN_BT	−28.1	2.58
	**DBT**	
BN/Ag-B_DBT	−29.1	2.60
BN/Ag-N_DBT	−24.4	2.62
BN/Ag-BN_DBT	−34.4	2.59
	**4,6-DMDBT**	
BN/Ag-B_DMDBT	−33.9	2.59
BN/Ag-N_DMDBT	−29.1	2.60
BN/Ag-BN_DMDBT	−39.2	2.58

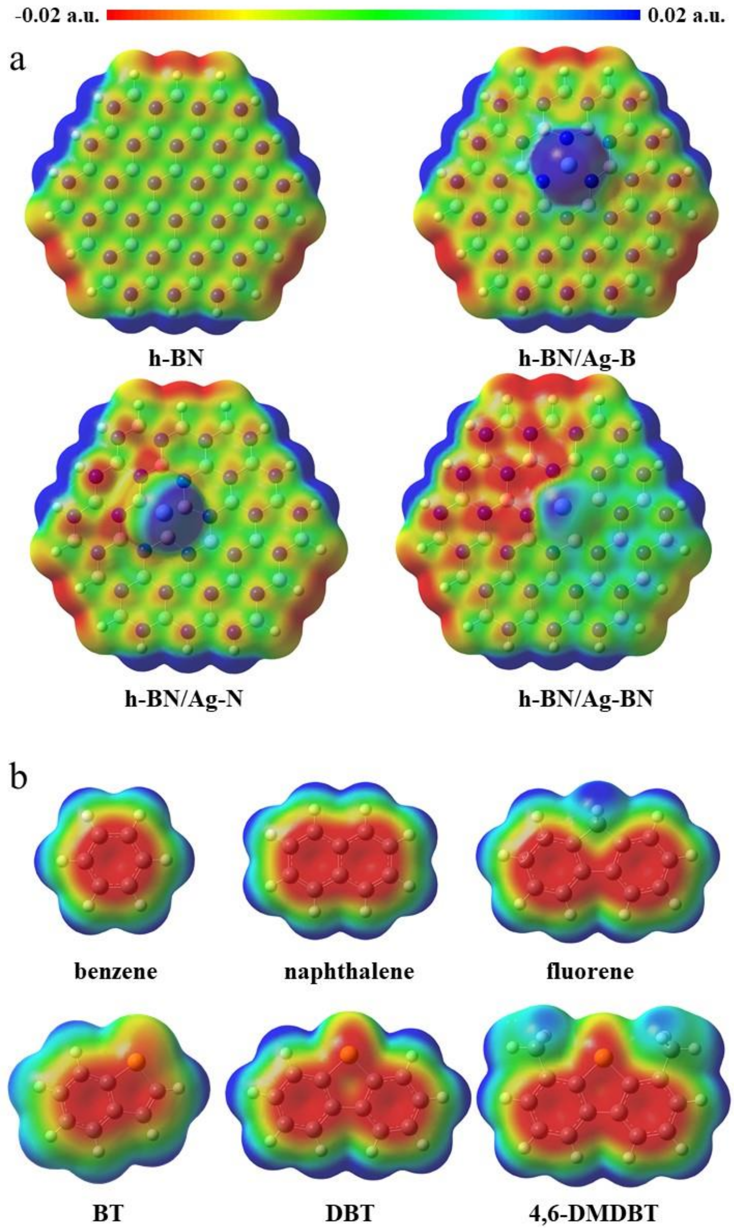

Figure S5. Electrostatic potential surface mapped on electron total density with an isovalue of 0.001. The colours range from −0.02 a.u. in red to 0.02 a.u. in blue for all the molecules. (**a**) The h-BN and the defective structures with the Ag embedded; (**b**) The aromatic compounds as absorbates.

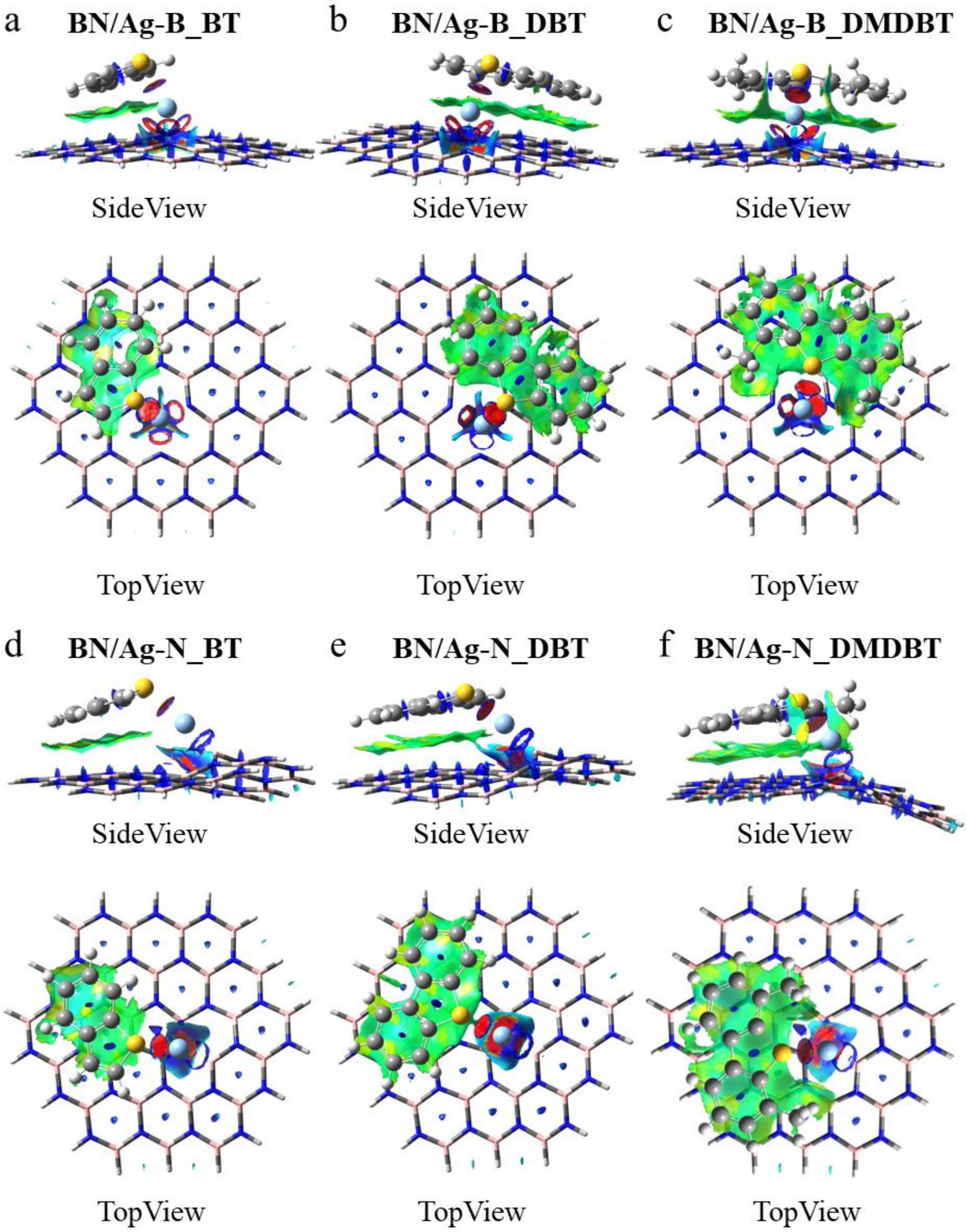

Figure S6. Gradient isosurfaces (s = 0.5 a.u.) of the BN/Ag-B and BN/Ag-N nanosheets for adsorption. The surfaces are colored on a red-green-blue scale according to values of sign $(\lambda_2)\varrho$, ranging from −0.02 to 0.02 a.u. (**a**) BN/Ag-B_BT, (**b**) BN/Ag-B_DBT, (**c**) BN/Ag-B_DMDBT, (**d**) BN/Ag-B_BT, (**e**) BN/Ag-B_DBT, (**f**) BN/Ag-B_DMDBT.
